# 1789. Comparison of influenza vaccine effectiveness estimates from the US Influenza Vaccine Effectiveness Network and Electronic Health Record Source Population Data, 2021-2022

**DOI:** 10.1093/ofid/ofad500.1618

**Published:** 2023-11-27

**Authors:** Callie McLean, Jessie R Chung, Manjusha Gaglani, MaryPatricia Nowalk, Emily T Martin, Sara Tartoff, Karen Wernli, Edward Belongia, Carlos G Grijalva, Brendan Flannery

**Affiliations:** Centers for Disease Control, Atlanta, Georgia; Centers for Disease Control and Prevention, Atlanta, GA; Baylor Scott & White Health, Temple, TX; University of Pittsburgh, Pittsburgh, Pennsylvania; University of Michigan, Ann Arbor, MI; KP Southern California, Pasadena, California; KP Washington, Seattle, Washington; Marshfield Clinic Research Institute, Marshfield, WI; Vanderbilt University Medical Center, Nashville, Tennessee; Centers for Disease Control and Prevention, Atlanta, GA

## Abstract

**Background:**

The COVID-19 pandemic led to increased respiratory viral testing for patients presenting with acute respiratory infection (ARI). Electronic health record (EHR) data from these visits can be used to assess influenza and COVID-19 vaccine effectiveness (VE). We compared VE data from participating healthcare institutions with VE against medically attended influenza among systematically tested patients actively enrolled in the US Flu VE Network.

**Methods:**

During the 2021-2022 influenza season, seven sites in the US Flu VE Network identified medically attended episodes of ARI in ambulatory settings based on ICD-10 diagnostic codes and clinical influenza and SARS-CoV-2 testing. Influenza status was determined from the clinical test results. Vaccination status was extracted from the EHR. During the same period, patients with ARI presenting at ambulatory clinics were actively enrolled and tested for influenza. VE and 95% confidence intervals (CI) were estimated comparing the odds of vaccination among influenza-positive versus influenza-negative patients using logistic regression models controlling for age, site, and calendar time.

**Results:**

During the 2021-2022 season, 220,162 medical visits for ARI with clinical testing were identified; 2% of tests were influenza positive, 26% were SARS-CoV-2 positive and 73% were negative for both. Overall, 34% of influenza positive and 42% of influenza negative patients had received influenza vaccines; VE using EHR data was 34% [95% CI 29-38]. Among 6,260 enrolled patients, 7% tested positive for influenza, 34% were SARS-CoV-2 positive and 62% were negative for both. Overall, 42% of influenza positive and 59% of influenza negative patients had received influenza vaccines. VE using enrolled patient data was 36% [95% CI 20-49]. VE using EHR data was 35%, 34%, and 6% for patients aged 6 months-17 years, 18-49, and 50+ years versus 40%, 32%, and 10% for enrolled patients.

**Conclusion:**

EHR based influenza VE estimates were similar to estimates from the US Flu VE study. Through continued widespread clinical testing, EHR-based platforms may present opportunities to assess VE in sub-groups with insufficient sample size in active surveillance.

**Disclosures:**

**MaryPatricia Nowalk, PhD, RDN**, Merck & Co.: Grant/Research Support|Merck & Co.: Honoraria|Sanofi: Grant/Research Support **Emily T. Martin, PhD, MPH**, Merck: Grant/Research Support **Edward Belongia, MD**, Seqirus: Grant/Research Support **Carlos G. Grijalva, MD, MPH**, AHRQ: Grant/Research Support|CDC: Grant/Research Support|FDA: Grant/Research Support|Merck: Advisor/Consultant|NIH: Grant/Research Support|Syneos Health: Grant/Research Support

